# Comparison Between Antigen and Allelic HLA Mismatches, and the Risk of Acute Rejection in Kidney Transplant Recipients

**DOI:** 10.1111/tan.70163

**Published:** 2025-04-07

**Authors:** Ryan Gately, Anne Taverniti, Narelle Watson, Armando Teixeira‐Pinto, Esther Ooi, Rowena Lalji, Ross Francis, Lucy Sullivan, William Mulley, Kate Wyburn, Scott Campbell, Carmel Hawley, Germaine Wong, Wai H. Lim

**Affiliations:** ^1^ Department of Kidney and Transplant Services Princess Alexandra Hospital Brisbane Queensland Australia; ^2^ Centre for Kidney Research, Kids Research Institute The Children's Hospital at Westmead Sydney New South Wales Australia; ^3^ New South Wales Transplantation and Immunogenetics Services Australian Red Cross Lifeblood Sydney Australia; ^4^ Sydney School of Public Health University of Sydney Sydney New South Wales Australia; ^5^ Medical School University of Western Australia Perth Western Australia Australia; ^6^ Department of Nephrology Queensland Children's Hospital Brisbane Australia; ^7^ South Australian Transplantation and Immunogenetics Service Australian Red Cross LifeBlood Adelaide South Australia Australia; ^8^ Department of Nephrology Monash Medical Centre Melbourne Australia; ^9^ Department of Medicine Monash University Melbourne Australia; ^10^ Department of Renal Medicine Royal Prince Alfred Hospital Sydney Australia; ^11^ Charles Perkins Centre Kidney Node University of Sydney Sydney Australia; ^12^ Department of Renal Medicine Westmead Hospital Sydney Australia; ^13^ Department of Renal Medicine and Transplantation Sir Charles Gairdner Hospital Perth Australia; ^14^ School of Medical and Health Sciences Edith Cowan University Perth Australia

**Keywords:** acute rejection, allelic mismatch, HLA antigens, kidney transplantation, predictive models, random survival forest

## Abstract

Deceased donor kidney allocation relies on HLA compatibility at the antigen level, as optimal matching reduces the risk of acute rejection. Whether HLA allele‐level mismatches improve, the prediction of acute rejection after transplantation remains unclear. Using data from the Australia and New Zealand Dialysis and Transplant Registry (ANZDATA) from 2017 to 2020, HLA antigenic and allelic mismatches between recipients and deceased donors were calculated with imputation of two‐field allelic equivalents undertaken where required. The discordance between antigen and allele mismatches was calculated, and oblique random survival forest models were used to predict acute rejection. Predictive performance of antigen (HLA‐A, ‐B, ‐DRB1 and ‐DQB1), allele (HLA‐A, ‐B, ‐DRB1 and ‐DQB1) and extended allele (HLA‐A, ‐B, ‐C, ‐DRB1, ‐DQA1 and ‐DQB1) models was examined using concordance index and integrated Brier scores, with variable importance calculated using permutation‐based methods. Among 2644 recipients followed for a median of 1.7 years, 521 recipients (20%) experienced acute rejection. Discordant numbers of antigenic and allelic mismatches occurred in 8%, 9%, 24% and 17% of HLA‐A, ‐B, ‐DRB1 and ‐DQB1 loci, respectively. Predictive performances were similar across all models, with concordance indices of 0.62–0.63 and integrated Brier scores of 0.09. HLA‐DRB1 and ‐DQB1 mismatches were the strongest predictors of acute rejection across models. In patients matched at the HLA‐DRB1 or ‐DQB1 antigen, those with allelic mismatches had similar incidences of rejection compared to those without. Allelic‐level assessment of HLA compatibility did not improve the prediction of acute rejection and may disadvantage certain recipients by reclassifying them into higher mismatch categories in allocation algorithms without providing clear clinical benefit.

## 1 Introduction

HLA matching is the standard immunological compatibility test for deceased donor kidney allocation worldwide, with optimal HLA matching reducing the risk of acute rejection after solid organ transplantation [1]. With the evolution of HLA typing from serological to deoxyribonucleic acid (DNA)‐sequence based typing methods, donor and recipient HLA gene polymorphism mismatches (also termed HLA allele mismatches) may provide a more accurate assessment of HLA immunological incompatibility compared to HLA antigen mismatches.

The availability of next‐generation sequencing (NGS) has enabled more efficient high‐resolution two‐field typing of the extended HLA genes with minimal ambiguity, although high‐resolution donor typing is often not available at the time of deceased donor kidney allocation because of the protracted turnaround time [2]. Although the assessment of allelic mismatches using high‐resolution HLA typing methods has improved the interpretation of the specificity of donor‐specific anti‐HLA antibody (DSA), it is unclear whether the inclusion of extended Classes I and II allelic mismatches improves the prediction of acute rejection after kidney transplantation beyond HLA‐A, ‐B and ‐DRB1 antigen mismatches. Furthermore, the impact on transplant potential from the incorrect assignment of the number of allelic HLA mismatches using low–intermediate resolution HLA typing at the time of deceased donor kidney allocation remains unknown.

In this study, we first examined the differences in the mismatch assignment between antigen and allelic levels at the HLA‐A, ‐B, ‐DRB1 and ‐DQB1 loci. We then evaluated whether allelic mismatches at HLA‐A, ‐B, ‐DRB1 and ‐DQB1 loci and across the extended loci improve the prediction of acute rejection, compared to antigen mismatches at these loci. In addition, we also determined which antigen and allelic mismatches are most predictive of acute rejection.

## 2 Methods

### 2.1 Study Cohort

We included all patients who received deceased donor kidney transplants in Australia between January 1, 2017, and December 31, 2020. Patients who received living‐donor kidney transplants and multi‐organ transplants were excluded. Ethics approval for the conduct of the study was granted by the Sir Charles Gairdner and Osborne Park Health Care Group Human Research Ethics Committee (RGS file number 2021/ET000826). The clinical and research activities being reported are consistent with the Principles of the Declaration of Istanbul as outlined in the ‘Declaration of Istanbul on Organ Trafficking and Transplant Tourism’.

### 2.2 Data Collection

Data were extracted from the Australia and New Zealand Dialysis and Transplant Registry (ANZDATA), which included: donor characteristics of age, sex, cause of death and comorbidities; recipient characteristics of age, sex, ethnicity, initial dialysis modality, dialysis duration, primary cause of kidney failure and comorbidities at time of transplant (diabetes, coronary artery disease, cerebrovascular disease and peripheral vascular disease) and transplant‐related characteristics of era, total ischaemic time, induction treatment, percentage calculated panel reactive antibody (cPRA) and presence/absence of pre‐transplant DSA. Donor and recipient HLA typing, and pre‐transplant DSA were extracted from OrganMatch, a clinical transplant online system that facilitates compatibility matching of donors and recipients for kidney transplantation. Pre‐transplant DSA positivity was defined as the ‘specific’ pre‐transplant anti‐HLA antibody directed against the donor antigen(s) recorded in OrganMatch, with mean fluorescence intensity (MFI) greater than 500 (where data were available).

### 2.3 Exposure

Donor and recipient molecular HLA typing at the antigen level (at one‐ or two‐field typing) at the time of donor kidney allocation was extracted from OrganMatch. We then calculated the number of antigen mismatches at HLA‐A, ‐B, ‐DRB1 and ‐DQB1, allele mismatches at HLA‐A, ‐B, ‐DRB1 and ‐DQB1, and extended allele mismatches at HLA‐A, ‐B, ‐C, ‐DRB1, ‐DQA and ‐DQB. We did not include mismatches at the HLA‐DRB3/4/5, ‐DPA and ‐DPB alleles for the calculation of extended HLA allele mismatches because some donors and recipients have no associated HLA‐DRB3/4/5 locus and/or had missing DP typing (up to 60%). Donor HLA typing was typically undertaken using molecular sequence‐specific oligonucleotide (SSO) and sequence‐specific primer (SSP) or with real‐time polymerase chain reaction (rtPCR) technologies at the time of deceased donor kidney allocation, while high‐resolution two‐field HLA typing was available for most transplant recipients. In cases with one‐field donor/recipient HLA typing, imputation to the two‐field equivalent was undertaken by a single experienced laboratory scientist using racial/ethnic group‐specific linkage equilibrium based on the catalogue of Common and Well Documented (CWD) alleles integrated into the Allele Frequencies Net Database (AFND) [3], Haplostats [4, 5] and the IMGT/HLA Database [6]. In cases without one‐field HLA antigen typing, two‐field HLA typing (where available) was converted to the antigen equivalent (for HLA‐A, ‐B, ‐DRB1 and ‐DQB1 antigens).

### 2.4 Outcome

The primary outcome was the time from transplant to any biopsy‐proven acute rejection, as reported to ANZDATA, including both cellular and antibody‐mediated rejection, until the end of the follow‐up period. Acute rejection episodes were recorded by clinicians or data managers of each hospital, according to the pre‐specified definitions in ANZDATA. Patients were censored at the end of the follow‐up period, or at the time of allograft loss or death, whichever occurred first. The number of discordant antigen and allele mismatches at the HLA‐A, ‐B, ‐DRB1 and ‐DQB1 loci was also calculated.

### 2.5 Statistical Methods

The baseline characteristics of the study cohort were reported as counts (proportions), means (standard deviation [SD]) or medians (interquartile range [IQR]) according to the distribution of the data and stratified by acute rejection status. An oblique random survival forest algorithm was used to create three models: (1) antigen mismatches at the HLA‐A, ‐B, ‐DRB1 and ‐DQB1 loci; (2) allele mismatches at the HLA‐A, ‐B, ‐DRB1 and ‐DQB1 loci and (3) allele mismatches at the HLA‐A, ‐B, ‐C, ‐DRB1, DQA1 and ‐DQB1 loci. Each of these models also included all donor, recipient and transplant‐related characteristics with less than 10% missing data. The predictive performance of each model was evaluated using the concordance index (*C*‐index), which measures the model's ability to discriminate between recipients with different risk levels, and the integrated Brier score, which reflects overall predictive accuracy. Both metrics were calculated over the entire follow‐up period, with the integrated Brier score incorporating inverse probability of censoring weighting (IPCW) to account for censoring. To reduce overfitting, both metrics were calculated using fivefold cross‐validation samples. Permutation‐based variable importance was calculated by independently shuffling each variable's values to assess its relative impact on predictive performance.

To improve clinical interpretability, we constructed confusion matrices for each of the three models displaying the number of true positives, true negatives, false positives and false negatives. From these values, we calculated each model's sensitivity, specificity, positive and negative predictive values for acute rejection within 12 months of transplantation. For these metrics, acute rejection was coded as a dichotomous variable, indicating the presence or absence of rejection within 12 months of transplant. Patients who died, suffered graft loss or were censored within 12 months of their transplant were excluded from this analysis. To determine the optimal probability for classifying recipients as having rejection or not, Youden's index was applied [7]. Youden's index identifies the threshold that maximises the sum of the sensitivity and specificity. Additionally, a category‐less Net Reclassification Index (NRI) at 12 months was calculated to determine whether either allelic model improved the classification of rejection compared to the base model (the antigen model).

With prior studies showing that the most important HLA mismatches in predicting acute rejection are HLA‐DRB1 and HLA‐DQB1 mismatches [1, 8], we next plotted cumulative incidence curves for acute rejection, accounting for the competing risks of allograft failure and death with a functioning allograft, stratified by donor/recipient pairs with discordant mismatches (i.e., matched at the antigen level but mismatched at the allele level) at the HLA‐DRB1 and ‐DQB1 loci. All analyses were undertaken using R version 4.4.0 (R Foundation for Statistical Computing, Vienna, Austria) [9]. The *missRanger* package was used to perform random forest imputation for variables with less than 10% missing data [10].

## 3 Results

### 3.1 Study Cohort

**FIGURE 1:**
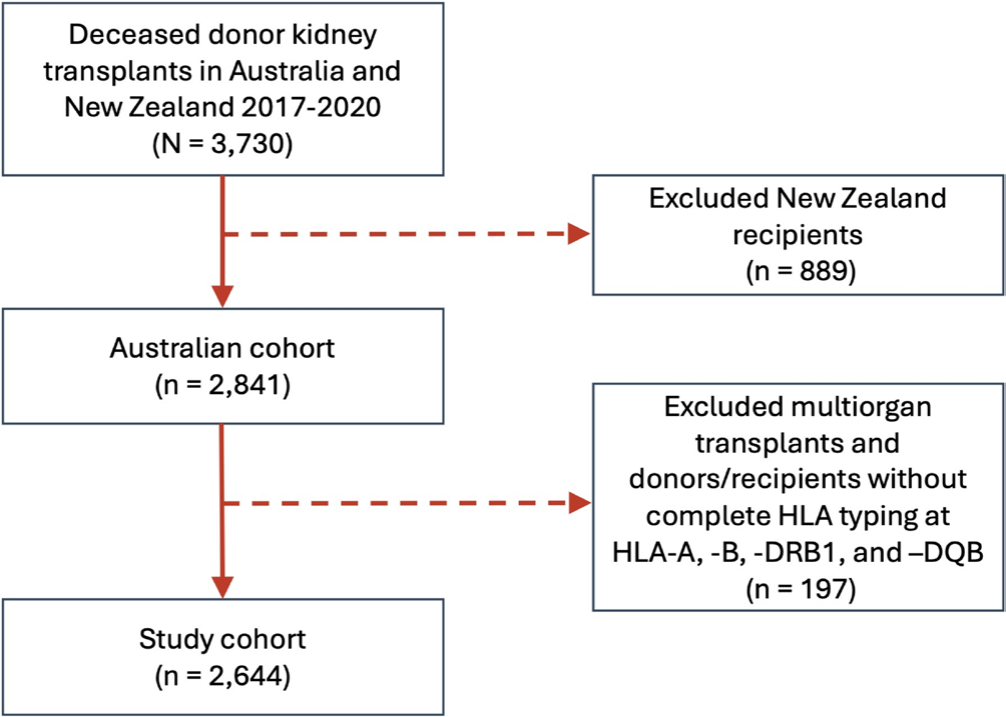
Flowchart of the study cohort.

Of 2841 patients who received deceased donor kidney transplants in Australia between January 2017 and December 2020, 197 patients (7%) were excluded due to missing donor or recipient HLA typing (at HLA‐A, ‐B and ‐DRB1) or because they were multi‐organ transplant recipients, leaving a study cohort of 2644 patients (Figure 1). Table S1 shows the number (%) of recipients and corresponding donors with imputed two‐field allele HLA typing for HLA‐A, ‐B, ‐C, ‐DRB1, ‐DQA1 and ‐DQB1.

The median age (IQR) of the study cohort was 55 (44–63) years, 36% were female, 69% of recipients received a deceased after brain death (DBD) kidney transplant, and 87% received a first allograft. Over a median (IQR) follow‐up of 1.7 (0.5–2.7) years, 521 (20%) experienced acute rejection, with 337 (65%) cases occurring in the first 3 months of transplantation. The respective median (IQR) follow‐up periods for those with and without rejection were 0.1 (0–0.4) and 2.0 (1.2–2.8) years. Table 1 shows the baseline donor, recipient and transplant‐related characteristics by acute rejection status. Recipients who experienced acute rejection were younger (median [IQR]: 52 [40, 62] vs. 55 [45, 63] years), more likely to have received a DCD kidney transplant (37% vs. 29%), and less likely to have received T‐cell depleting induction treatment (7% vs. 11%) compared to recipients without acute rejection. The proportion of recipients with pre‐transplant DSAs was not different between groups, and the median number (IQR) of HLA‐A, ‐B, ‐DRB1 and ‐DQB1 antigen and allele mismatches was 6 [3, 4, 5, 6, 7] and 7 [4, 5, 6, 7].

**TABLE 1:** Baseline characteristics of the study cohort by acute rejection status.

Characteristics	Overall, *N* = 2,644^a^	No rejection, *N* = 2,123^a^	Rejection, *N* = 521^a^	*p* ^b^
Median follow‐up	1.7 (0.5, 2.7)	2.0 (1.2, 2.8)	0.1 (0.0, 0.4)	**< 0.001**
*Recipient factors*				
Sex				0.2
M	1679 (64%)	1336 (63%)	343 (66%)	
F	965 (36%)	787 (37%)	178 (34%)	
Age	55 (44, 63)	55 (45, 63)	52 (40, 62)	**0.002**
Ethnicity				0.082
Caucasian	1661 (66%)	1319 (66%)	342 (69%)	
ATSI	163 (6.5%)	136 (6.8%)	27 (5.4%)	
Pasifika	94 (3.8%)	69 (3.4%)	25 (5.0%)	
Other	149 (5.9%)	117 (5.8%)	32 (6.4%)	
Asian	438 (17%)	367 (18%)	71 (14%)	
Missing	139	115	24	
Blood group				0.4
A	1018 (39%)	822 (39%)	196 (38%)	
O	1255 (47%)	998 (47%)	257 (49%)	
B	276 (10%)	222 (10%)	54 (10%)	
AB	94 (3.6%)	81 (3.8%)	13 (2.5%)	
Missing	1	0	1	
BMI	26.7 (22.9, 30.7)	26.6 (22.8, 30.5)	27.1 (23.2, 31.3)	**0.023**
Missing	81	66	15	
Graft number				0.2
1	2294 (87%)	1,854 (87%)	440 (84%)	
2	292 (11%)	227 (11%)	65 (12%)	
3	53 (2.0%)	38 (1.8%)	15 (2.9%)	
4	5 (0.2%)	4 (0.2%)	1 (0.2%)	
Smoking status				0.8
Never	1497 (58%)	1199 (58%)	298 (59%)	
Current/former	1076 (42%)	867 (42%)	209 (41%)	
Missing	71	57	14	
Coronary artery disease				0.3
N	2068 (78%)	1651 (78%)	417 (80%)	
S	0 (0%)	0 (0%)	0 (0%)	
Y	567 (22%)	464 (22%)	103 (20%)	
Missing	9	8	1	
Peripheral vascular disease				0.9
N	2321 (88%)	1864 (88%)	457 (88%)	
S	0 (0%)	0 (0%)	0 (0%)	
Y	314 (12%)	251 (12%)	63 (12%)	
Missing	9	8	1	
Cerebrovascular disease				0.077
N	2479 (94%)	1981 (94%)	498 (96%)	
S	0 (0%)	0 (0%)	0 (0%)	
Y	156 (5.9%)	134 (6.3%)	22 (4.2%)	
Missing	9	8	1	
Diabetes				0.7
No	1843 (70%)	1481 (70%)	362 (69%)	
T1DM	77 (2.9%)	64 (3.0%)	13 (2.5%)	
T2DM	716 (27%)	570 (27%)	146 (28%)	
Missing	8	8	0	
Initial dialysis modality				**0.005**
HD	1560 (61%)	1225 (59%)	335 (66%)	
PD	1010 (39%)	839 (41%)	171 (34%)	
Missing	74	59	15	
mPRA	0 (0, 12)	0 (0, 9)	0 (0, 28)	0.2
Primary kidney disease				0.2
Diabetic kidney disease	532 (20%)	427 (20%)	105 (20%)	
Glomerulonephritis	955 (36%)	757 (36%)	198 (38%)	
Hypertension	193 (7.3%)	162 (7.6%)	31 (6.0%)	
Polycystic disease	311 (12%)	253 (12%)	58 (11%)	
Reflux nephropathy	142 (5.4%)	105 (4.9%)	37 (7.1%)	
Other	511 (19%)	419 (20%)	92 (18%)	
*Donor factors*				
Donor age	49 (35, 60)	49 (35, 60)	49 (37, 58)	0.9
Donor sex				0.7
F	1098 (42%)	886 (42%)	212 (41%)	
M	1546 (58%)	1237 (58%)	309 (59%)	
Donor diabetes				0.10
No	2441 (92%)	1951 (92%)	490 (94%)	
T2DM	203 (7.7%)	172 (8.1%)	31 (6.0%)	
Donor hypertension				0.9
Y	661 (25%)	533 (25%)	128 (25%)	
N	1962 (75%)	1576 (75%)	386 (75%)	
Missing	21	14	7	
Donor smoking status				0.9
Current/former	1712 (65%)	1373 (65%)	339 (65%)	
Never	924 (35%)	743 (35%)	181 (35%)	
Missing	8	7	1	
Donor history of cancer				> 0.9
N	2420 (92%)	1942 (92%)	478 (92%)	
Y	216 (8.2%)	173 (8.2%)	43 (8.3%)	
Missing	8	8	0	
Circulatory vs. brain death				**< 0.001**
DBD	1829 (69%)	1500 (71%)	329 (63%)	
DCD	815 (31%)	623 (29%)	192 (37%)	
*Transplant factors*				
Year of transplant				**< 0.001**
2017	670 (25%)	499 (24%)	171 (33%)	
2018	829 (31%)	662 (31%)	167 (32%)	
2019	797 (30%)	665 (31%)	132 (25%)	
2020	348 (13%)	297 (14%)	51 (9.8%)	
Total ischaemic time	10.0 (7.0, 13.0)	10.0 (7.0, 13.0)	9.0 (7.0, 13.0)	0.9
Missing	93	78	15	
Induction with anti‐CD25	2203 (83%)	1745 (82%)	458 (88%)	**0.002**
Induction with T‐cell depletion	273 (10%)	236 (11%)	37 (7.1%)	**0.007**
Pre‐transplant DSA	162 (6.5%)	132 (6.5%)	30 (6.1%)	0.7
Missing	136	105	31	

*Note:* Bold values indicated statistically significant results i.e., *p*‐values < 0.05.

^a^ Median (Q1, Q3); *n* (%).

^b^ Wilcoxon rank sum test; Pearson's chi‐squared test; Fisher's exact test.

### 3.2 Discordant Antigen and Allele Mismatches

The discordant antigen and allele mismatches at the HLA‐A‐, ‐B, ‐DRB1 and ‐DQB1 loci are shown in Table 2. A discordant number of antigenic and allelic mismatches occurred in 8%, 9%, 24% and 17% of HLA‐A, ‐B, ‐DRB1 and ‐DQB1 loci. For HLA‐A, an identical number of antigen and allele mismatches was observed in 2439 (92%) cases. In 446 recipients with 0 HLA‐A antigen mismatches, 57 (13%) and 5 (1%) had one and two allele mismatches, respectively. For HLA‐B, an identical number of antigen and allele mismatches was observed in 2402 (91%) cases. In 970 recipients with one HLA‐B antigen mismatch, 171 (18%) had two allele mismatches. For HLA‐DRB1 and HLA‐DQB1, identical numbers of antigen and allele mismatches were observed in 2019 (76%) and 2025 (77%) of cases, respectively. Of those with one HLA‐DRB1 antigen mismatch, 320 (35%) had two allele mismatches. Of those with one HLA‐DQB1 antigen mismatch, 377 (30%) had two allele mismatches.

**TABLE 2:** Discordant HLA antigenic and allelic mismatches.

	HLA allelic mismatches		
	0	1	2
HLA‐A antigenic mismatches			
0	382 (86.0)	57 (12.9)	5 (1.1)
1	0 (0.0)	1078 (88.3)	143 (11.7)
2	0 (0.0)	0 (0.0)	979 (100.0)
HLA‐B antigenic mismatches			
0	228 (76.3)	65 (21.7)	6 (2.0)
1	0 (0.0)	799 (82.4)	171 (17.6)
2	0 (0.0)	0 (0.0)	1375 (100.0)
HLA‐DRB1 antigenic mismatches			
0	374 (55.1)	248 (36.5)	57 (8.4)
1	0 (0.0)	582 (64.5)	320 (35.5)
2	0 (0.0)	0 (0.0)	1063 (100.0)
HLA‐DQB1 antigenic mismatches			
0	459 (65.4)	199 (28.4)	43 (6.2)
1	0 (0.0)	876 (69.9)	377 (30.1)
2	0 (0.0)	0 (0.0)	690 (100.0)

*Note:* Data expressed as number (proportion).

### 3.3 Prediction of Acute Rejection of HLA Antigen and Allele Mismatches

**FIGURE 2:**
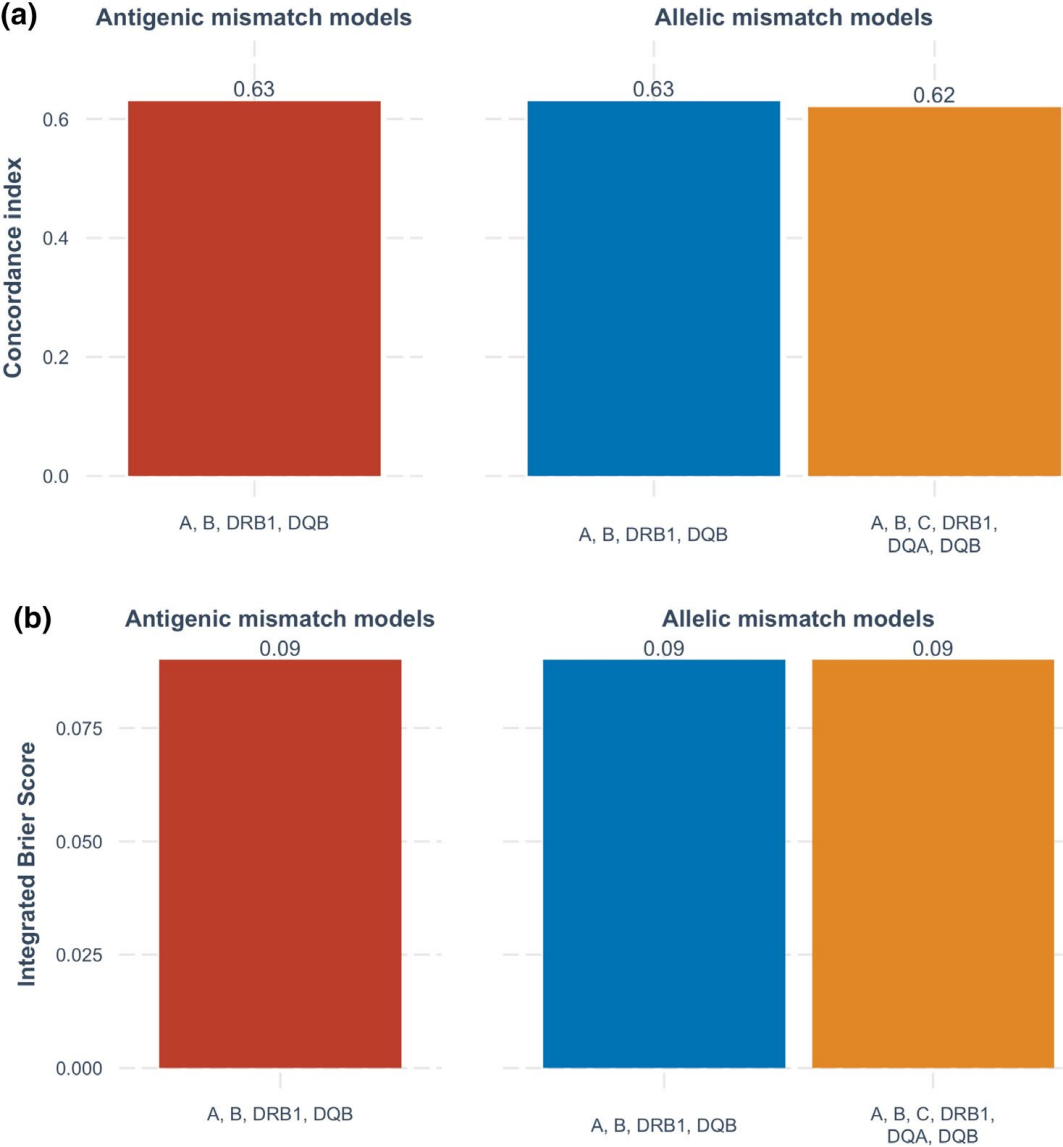
(a) Predictive performance of models for acute rejection based on *C*‐index over the complete follow‐up period. (b) Predictive performance of models for acute rejection based on integrated Brier score over the complete follow‐up period.

The respective *C*‐indices and integrated Brier scores for the acute rejection models based on HLA antigen, HLA allele and extended HLA allele mismatches are shown in Figure 2a,b. All three models showed moderate and very similar predictive performance, with *C*‐indices of 0.63, 0.63 and 0.62 for the HLA antigen‐, HLA allele‐ and extended HLA allele‐mismatched models respectively. The integrated Brier scores were also similar, each measuring 0.09.

### 3.4 Variable Importance for Predicting Acute Rejection

**FIGURE 3:**
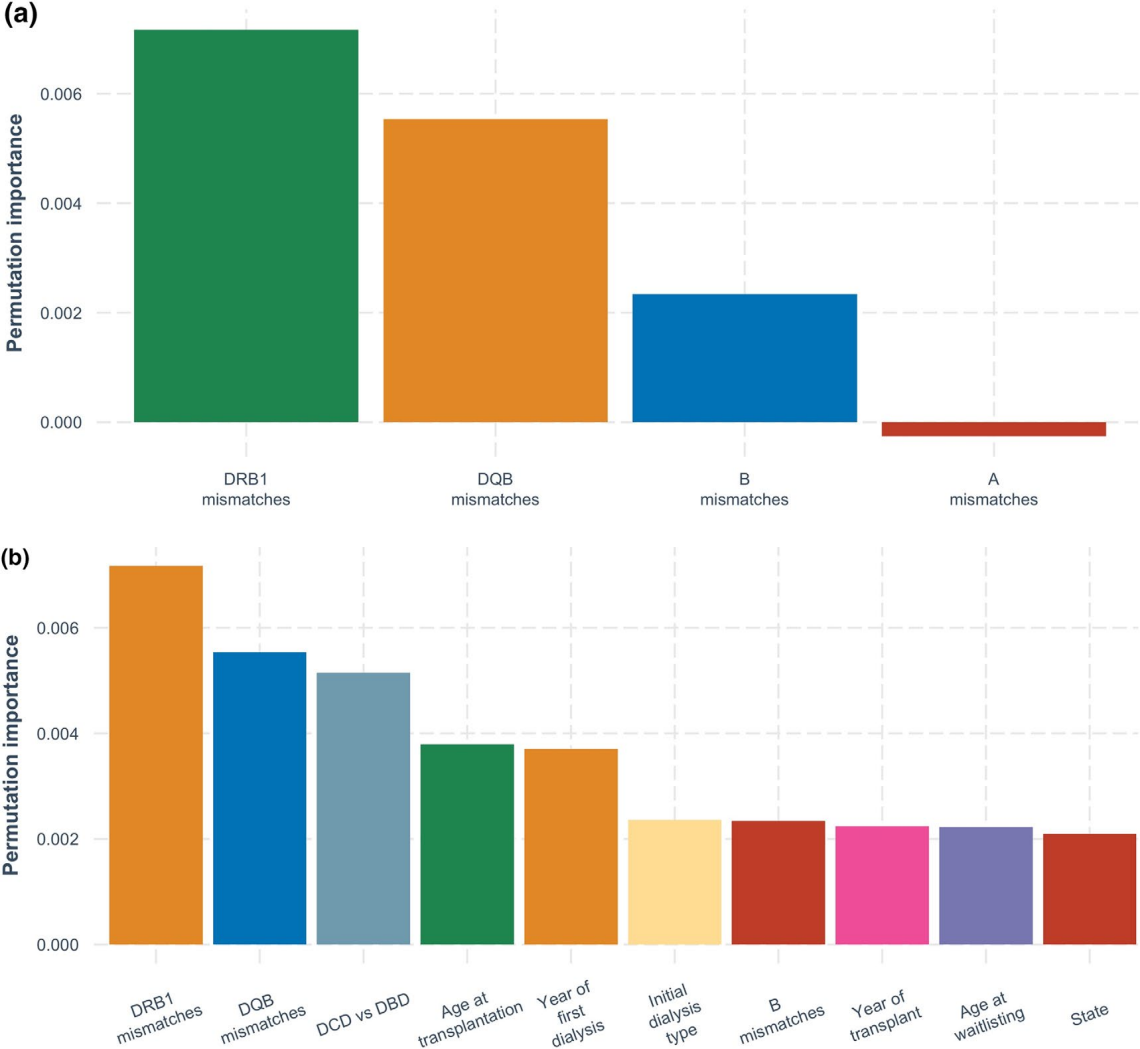
(a) Permutation‐based variable importance for the antigen model over the complete follow‐up period (showing HLA mismatch variables only). (b) Permutation‐based variable importance for the antigen model over the complete follow‐up period (showing 10 most important variables overall).

**FIGURE 4:**
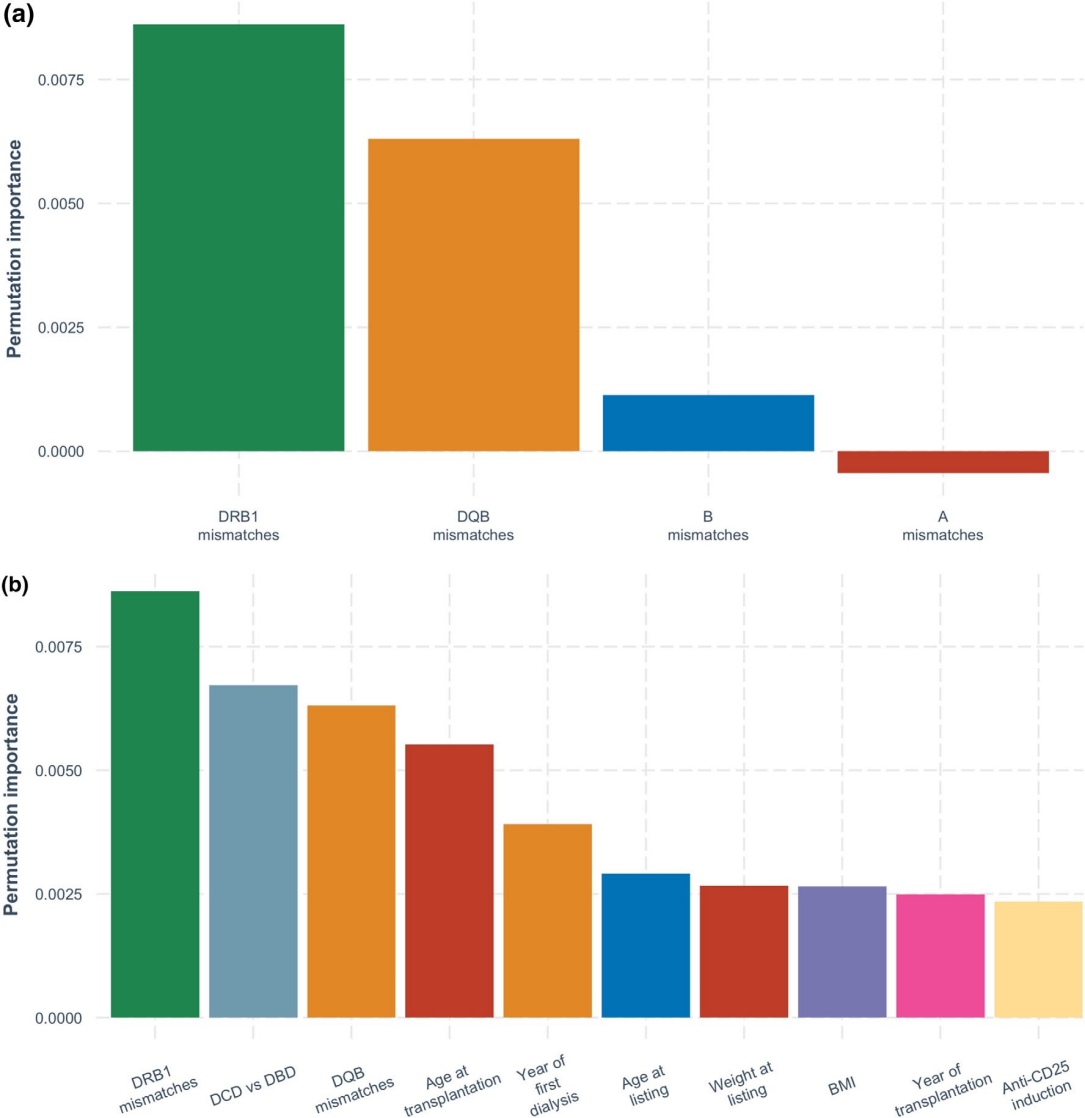
(a) Permutation‐based variable importance for the allele model over the complete follow‐up period (showing HLA mismatch variables only). (b) Permutation‐based variable importance for the allele model over the complete follow‐up period (showing 10 most important variables overall).

**FIGURE 5:**
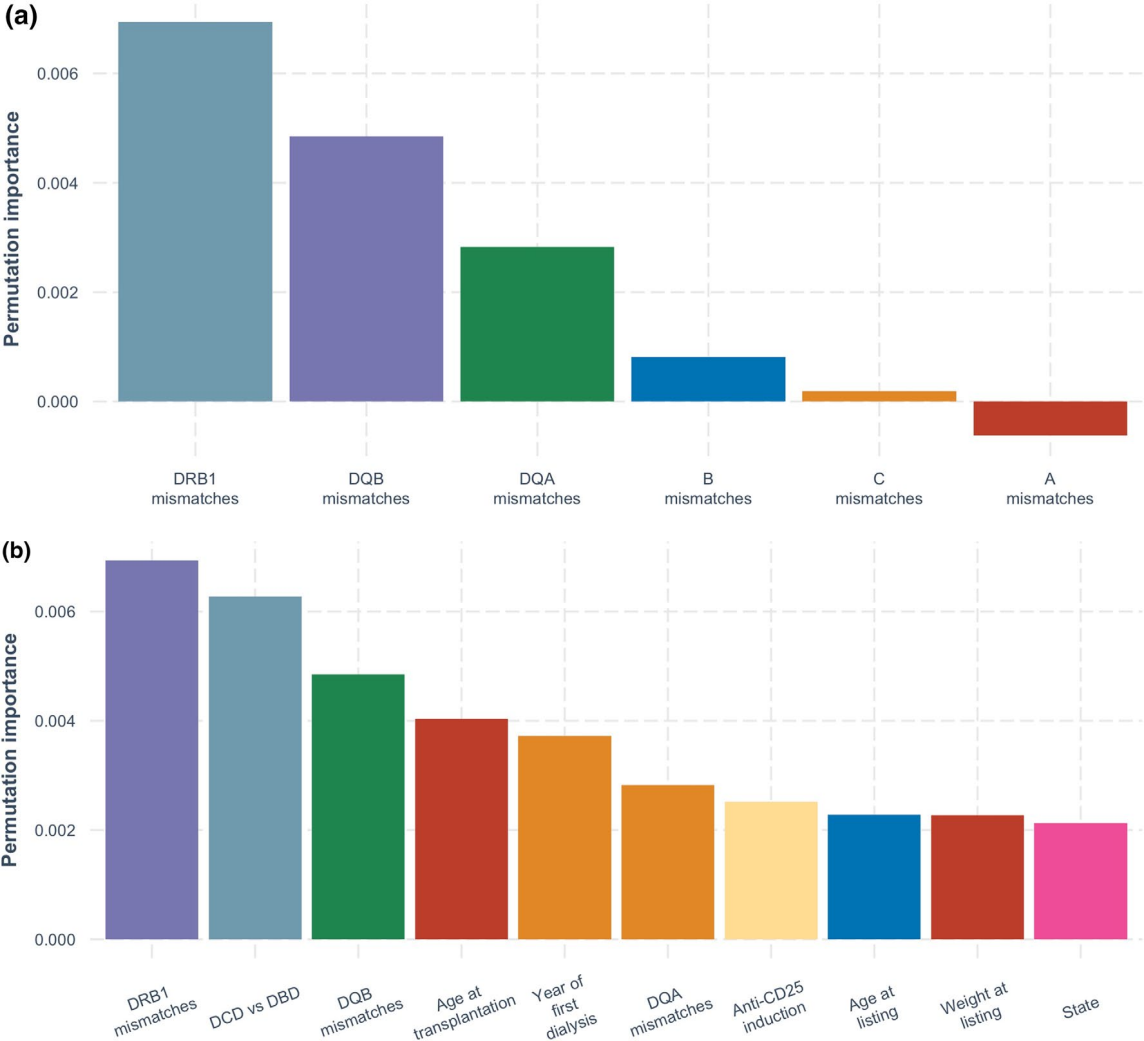
(a) Permutation‐based variable importance for the extended allele model over the complete follow‐up period (showing HLA mismatch variables only). (b) Permutation‐based variable importance for the extended allele model over the complete follow‐up period (showing 10 most important variables overall).

In the antigen model, the most important variables for predicting acute rejection were HLA‐DRB1 mismatches, followed by HLA‐DQB1 mismatches and donor type (DCD vs. DBD) (Figure 3a,b). In both the allelic and extended allelic models (Figures 4a,b and 5a,b), the most important variable was HLA‐DRB1 mismatches, followed by donor type (DCD vs. DBD), HLA‐DQB1 mismatches and recipient age at transplantation.

### 3.5 Classification Metrics

A total of 2270 patients were included in the calculation of classification metrics. The confusion matrices for each model are shown in Tables S2–S4. Youden's index for the antigen, allele and extended allele models was 0.78, 0.79 and 0.76, respectively. Amongst the three models, the antigen model demonstrated the highest sensitivity (0.9 [95% CI, 0.87–0.92]), specificity (0.89 [95% CI, 0.87–0.92]), positive predictive value (0.67 [95% CI, 0.63–0.7]) and negative predictive value (0.97 [95% CI, 0.96–0.98]) for classifying rejection within 12 months of transplant. Compared to the antigen model, the category‐less NRI for the allelic model was −0.091 (95% CI, −0.123 to −0.060) and for the extended allelic model was −0.015 (95% CI, −0.037 to 0.009), indicating that neither model improved the classification of rejection within 12 months of transplant.

### 3.6 Cumulative Incidence of Acute Rejection in Recipients of HLA‐DRB1 and ‐DQB1 Antigen Matched but Allele‐Mismatched Transplants

**FIGURE 6:**
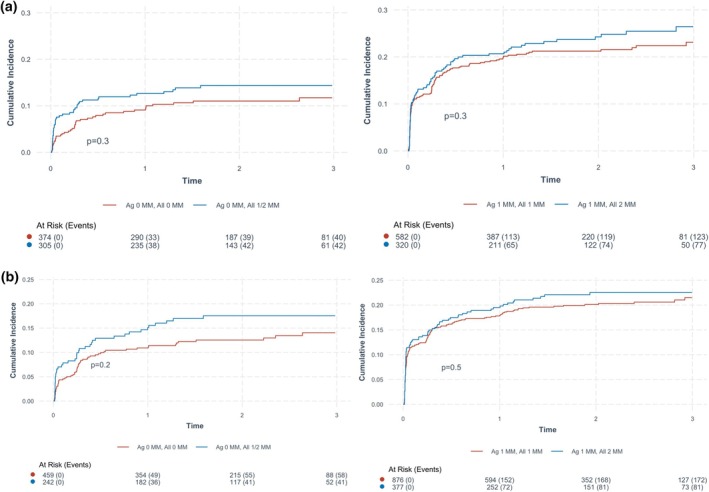
(a) Cumulative incidence of rejection in DRB1 discordant pairs with competing risks of death and graft loss. (b) Cumulative incidence of rejection in DQB1 discordant pairs with competing risks of death and graft loss.

Figure 6a,b shows the cumulative incidence curves for discordant HLA‐DRB1 and HLA‐DQB1 antigen/allele mismatched cases, adjusted for the competing risks of allograft loss and death with a functioning allograft. Among recipients who received 0 HLA‐DRB1 antigen mismatched kidneys, the cumulative incidence of acute rejection was similar between those with and without HLA‐DRB1 allele mismatches (*p* = 0.3). For recipients who received 1 HLA‐DRB1 antigen mismatched kidney, the cumulative incidence of acute rejection was also similar between those with 1 or > 1 allelic mismatches (*p* = 0.3). Similar findings were observed for recipients who received 0 and 1 HLA‐DQB1 antigen mismatched kidneys.

## 4 Discussion

Donor kidney allocation continues to rely primarily on HLA antigen matching between donors and potential kidney transplant candidates. Advances in the field of histocompatibility have allowed transplant programmes to define HLA compatibility at the allelic level, but the impact of allelic mismatches on kidney allograft outcome remains poorly defined. In this contemporary cohort of transplant recipients of deceased donor kidneys, models that included clinical variables and HLA antigenic and allelic mismatches showed moderate predictive performance, with mismatches at the HLA‐DRB1 and ‐DQB1 loci being the most important for predicting acute rejection. The test performance of the HLA antigen and HLA allele mismatched models was similar, with the HLA antigen mismatched model conferring the best sensitivity and specificity in predicting acute rejection. The inclusion of matching at the extended alleles did not improve prediction performance for acute rejection. There were high numbers of discordant antigenic and allelic mismatches at the HLA‐DRB1 and ‐DQB1 loci, although this did not translate into a difference in the incidence of acute rejection rates. These findings suggest that the consideration of HLA allelic matches did not improve the prediction of acute rejection over traditional HLA antigenic mismatches in deceased donor kidney transplantation.

Current allocation practice in most countries, including Australia, the United States, Canada and the United Kingdom, considers HLA antigen matching at the HLA‐A, ‐B, ‐DRB1 ± ‐DQB1 loci in prioritising allocation of better HLA antigen matched donor kidneys to potential kidney transplant candidates. From the early 2000s, molecular methods based on DNA‐based molecular methods for HLA allele typing have largely replaced serological typing for deceased donors in most HLA laboratories. For deceased donor kidney allocation, HLA allele typing is converted back to the serologically antigen equivalent during organ allocation, although it is plausible (but extremely infrequent) that certain alleles have no determined serologically defined antigens.

In kidney transplantation, incremental antigenic mismatches at HLA‐A, ‐B, ‐DRB1 and ‐DQB1 are consistently associated with a greater risk of allo‐sensitisation, acute rejection and/or allograft failure [1, 8, 11, 12, 13, 14]. Although low‐to‐intermediate one‐field resolution donor allele‐level HLA typing is readily available at the time of deceased donor kidney allocation, this method will only provide sequence information for a group of alleles that are encoded by a specific antigen or sequence homology to a specific allele. Often, the availability of high‐resolution HLA sequencing resolves most of the HLA typing ambiguities, especially at the Class II alleles. Practically, the availability of two‐field allele‐level donor typing is often essential in assisting HLA scientists and clinicians to distinguish between the alleles of interest when a candidate has an allele‐specific antibody, a critical component of performing accurate virtual crossmatch assessment prior to transplantation. The availability of rapid high‐resolution donor HLA typing using Nanopore sequencing technology may provide this information to inform donor kidney allocation within a short turnaround time and will be able to clearly define allelic mismatches across the extended alleles [15, 16, 17]. However, the associations between allelic mismatches and other hard allograft outcomes, such as acute rejection or allograft failure in kidney transplantation, and whether this has greater predictive capacity than antigenic mismatches for these outcomes remain poorly defined.

The clinical importance of HLA allele‐level compatibility, including HLA‐DQ matching, is well established in haematopoietic stem cell transplantation for haematological malignancies. It improves donor selection, reduces engraftment failure and allo‐sensitisation risk, and enhances risk stratification for graft‐versus‐host disease, all‐cause mortality and non‐relapse mortality, even in serologically matched transplants [18, 19, 20, 21, 22, 23, 24, 25, 26].

In contrast to the findings in umbilical cord and haematopoietic stem cell transplants, our study demonstrates that HLA antigen‐level mismatches have a predictive ability similar to that of HLA allele‐level mismatches, with HLA‐DRB1 and ‐DQB1 antigenic or allelic mismatches emerging as the most important HLA types to predict acute rejection. Patients who received kidneys that were antigen‐matched but allele‐mismatched at HLA‐DRB1 or HLA‐DQB1 demonstrated cumulative incidences of acute rejection comparable to those observed in patients with identical numbers of antigen‐ and allele‐level mismatches at these loci. As each HLA antigen encodes for many different alleles, single allele‐level incompatibility (i.e., less disparity in differences in amino acid residues) may be less likely to achieve the threshold required for a more robust T‐ and B‐cell immune responses, a plausible explanation of why allele‐level matching does not improve the predictive capacity for acute rejection [27].

With a greater characterisation of the HLA structure and compatibility with more advanced histocompatibility techniques, there is considerable interest in integrating HLA molecular matching in place of HLA antigen matching in deceased donor kidney allocation schemes to achieve the allocation of better HLA compatible donor kidneys to potential kidney transplant candidates. In fact, an allocation scheme that explicitly considers HLA epitope mismatches over HLA antigen mismatches has been implemented in the allocation of donor kidneys to paediatric and adolescent transplant recipients, with acceptable short‐term outcomes and conceivably lower risks of allo‐sensitisation compared to historical controls [28]. Given the large number of epitopes expressed by each HLA molecule, and the difficulty in defining clinically relevant epitope (or eplet) mismatch thresholds, allocation schemes based solely on epitope mismatches may be challenging to implement. While HLA allele‐level matching to select the best unrelated donors for haematopoietic cell transplantation has been supported, utilising allelic mismatches in the donor kidney allocation scheme is not readily accepted, in part due to the lack of data supporting this approach.

Our study showed that the calculation of allele‐level mismatches resulted in reassignment into a higher HLA mismatch category in 45% of recipients. Consequently, this classification could have led to the reallocation of the intended donor kidney to another patient on the waitlist, potentially prolonging waiting time for these recipients. Given the prediction of acute rejection after kidney transplantation was not improved using allele‐level mismatches, the integration of HLA allele mismatches into an allocation algorithm could disadvantage a subset of recipients without providing any clear clinical benefit. However, it must be emphasised that differences in the prediction of allo‐sensitisation between HLA antigenic and allelic mismatches cannot be established, as data relating to the development de novo DSA were not captured by ANZDATA.

The strengths of this study include the completeness of the dataset, the availability of molecular HLA typing for both donors and recipients and the accurate ascertainment of the outcome measure of acute rejection after kidney transplantation. However, there are several notable limitations that are inherent in registry studies. There are likely residual and unmeasured confounders not captured in ANZDATA, such as the systematic variations in the acceptance of donor kidneys for transplantation, management of patients with varying immunological risk profiles, cumulative exposure tof immunosuppressive medications, lack of therapeutic drug levels and potential differences in the practice of protocol and indication biopsies, which may have modified our study findings. Furthermore, a proportion of donor and/or recipient HLA allele typing was imputed, especially for HLA‐DQA1 and DQB1 alleles, the inaccuracy of which may result in ascertainment bias of the true number of allelic mismatches [29]. However, the imputation was undertaken by an experienced HLA scientist, and most donors and recipients were Caucasians, therefore reducing the error rates of incorrect imputation of donor and recipient HLA alleles.

Assessment of HLA compatibility at the allele level does not improve the prediction of acute rejection after kidney transplantation, compared to HLA antigen‐level assessment. Moreover, it may disadvantage some recipients by assigning them a higher number of HLA mismatches, thereby reducing their transplant potential. Although not evaluated in this study, consideration of allele‐specific mismatch(es) remains important in donor kidney allocation, as it enables more accurate identification of pre‐transplant anti‐HLA antibody specificity and future allo‐sensitisation risk.

## Conflicts of Interest

The authors declare no conflicts of interest.

## Supporting information


**Data S1.** Supporting Information.

## Data Availability

The data used in this study were obtained from the Australia and New Zealand Dialysis and Transplant Registry (ANZDATA). Access to ANZDATA is restricted and subject to governance and ethical approval. The data are not publicly available due to confidentiality and privacy restrictions.
