# Supplementary material for: Comparison Between Antigen and Allelic HLA Mismatches, and the Risk of Acute Rejection in Kidney Transplant Recipients
Source: HLA. 2025 Apr 7;105(4):e70163. doi: 10.1111/tan.70163 (PMC11975158; doi:10.1111/tan.70163)
Supplement: Supplementary file 1 — Data S1. Supporting Information. [file TAN-105-e70163-s001.docx]

Table S1. Donors and recipients with imputed 2-field allele HLA typing.

| **HLA locus** | **Donor (n=1,664)** | **Recipient (n=2,644)** |
| --- | --- | --- |
| **HLA-A** | 308 (19%) | 1,181 (44%) |
| **HLA-B** | 314 (19%) | 874 (33%) |
| **HLA-C** | 717 (43%) | 1,453 (55%) |
| **HLA-DRB1** | 323 (19%) | 777 (29%) |
| **HLA-DQA1** | 69 (4%) | 1,006 (38%) |
| **HLA-DQB1** | 516 (31%) | 1,289 (49%) |

*Data expressed as counts and percentages. HLA – human leukocyte antigen.*

Table S2. Confusion matrix for rejection within 12 months of transplant using the antigen model.

|  | **Predicted Positive** | **Predicted Negative** |  |
| --- | --- | --- | --- |
| **Actual Positive** | 413 (TP) | 46 (FN) | Sens: 0.9 (0.87-0.92) |
| **Actual Negative** | 208 (FP) | 1603 (TN) | Spec: 0.89 (0.87-0.9) |
|  | PPV: 0.67 (0.63-0.7) | NPV: 0.97 (0.96-0.98) |  |
| *TP = True Positives, TN = True Negatives, FP = False Positives, FN = False Negatives, Sens = Sensitivity (95% CI), Spec = Specificity (95% CI), PPV = Positive predictive value (95% CI), NPV = Negative predictive value (95% CI)* | | | |
|  |  |  |  |

Table S3. Confusion matrix for rejection within 12 months of transplant using the allele model.

|  | **Predicted Positive** | **Predicted Negative** |  |
| --- | --- | --- | --- |
| **Actual Positive** | 405 (TP) | 54 (FN) | Sens: 0.88 (0.85-0.91) |
| **Actual Negative** | 334 (FP) | 1477 (TN) | Spec: 0.82 (0.8-0.83) |
|  | PPV: 0.55 (0.51-0.58) | NPV: 0.96 (0.95-0.97) |  |
| *TP = True Positives, TN = True Negatives, FP = False Positives, FN = False Negatives, Sens = Sensitivity (95% CI), Spec = Specificity (95% CI), PPV = Positive predictive value (95% CI), NPV = Negative predictive value (95% CI)* | | | |
|  |  |  |  |

Table S4. Confusion matrix for rejection within 12 months of transplant using the extended allele model.

|  | **Predicted Positive** | **Predicted Negative** |  |
| --- | --- | --- | --- |
| **Actual Positive** | 407 (TP) | 52 (FN) | Sens: 0.89 (0.85-0.91) |
| **Actual Negative** | 211 (FP) | 1600 (TN) | Spec: 0.88 (0.87-0.9) |
|  | PPV: 0.66 (0.62-0.69) | NPV: 0.97 (0.96-0.98) |  |
| *TP = True Positives, TN = True Negatives, FP = False Positives, FN = False Negatives, Sens = Sensitivity (95% CI), Spec = Specificity (95% CI), PPV = Positive predictive value (95% CI), NPV = Negative predictive value (95% CI)* | | | |
|  |  |  |  |
